# Central and Peripheral Mechanisms in ApoE4-Driven Diabetic Pathology

**DOI:** 10.3390/ijms21041289

**Published:** 2020-02-14

**Authors:** Amit Koren-Iton, Shiran Salomon-Zimri, Alex Smolar, Efrat Shavit-Stein, Amir Dori, Joab Chapman, Daniel M. Michaelson

**Affiliations:** 1 Department of Neurobiology, The Sagol School of Neuroscience, The George S. Wise Faculty of Life Sciences, Tel-Aviv University, Tel Aviv 6997801, Israel; amitkoren92@gmail.com (A.K.-I.); shiransalmon@gmail.com (S.S.-Z.); nakaryak@gmail.com (A.S.); 2 Department of Neurology, The Chaim Sheba Medical Center, Ramat Gan 5261, Israel; efrat.shavit.stein@gmail.com (E.S.-S.); amir.dori@gmail.com (A.D.); jchapman@tauex.tau.ac.il (J.C.); 3 Department of Neurology and Neurosurgery, Sackler Faculty of Medicine, Tel Aviv University, Tel Aviv 6997801, Israel; 4 Department of Physiology and Pharmacology, Sackler Faculty of Medicine, Tel Aviv University, Tel Aviv 6997801, Israel; 5 Robert and Martha Harden Chair in Mental and Neurological Diseases, Sackler Faculty of Medicine, Tel Aviv University, Tel Aviv 6997801, Israel

**Keywords:** Alzheimer’s disease (AD), apolipoprotein E4 (apoE4), type 2 diabetes mellitus (T2DM), targeted replacement (TR) mice, high-fat diet (HFD), hippocampus, insulin signaling, insulin resistance

## Abstract

Apolipoprotein E (*APOE*) ε4 gene allele and type 2 diabetes mellitus (T2DM) are prime risk factors for Alzheimer’s disease (AD). Despite evidence linking T2DM and apoE4, the mechanism underlying their interaction is yet to be determined. In the present study, we employed a model of *APOE*-targeted replacement mice and high-fat diet (HFD)-induced insulin resistance to investigate diabetic mechanisms associated with apoE4 pathology and the extent to which they are driven by peripheral and central processes. Results obtained revealed an intriguing pattern, in which under basal conditions, apoE4 mice display impaired glucose and insulin tolerance and decreased insulin secretion, as well as cognitive and sensorimotor characteristics relative to apoE3 mice, while the HFD impairs apoE3 mice without significantly affecting apoE4 mice. Measurements of weight and fasting blood glucose levels increased in a time-dependent manner following the HFD, though no effect of genotype was observed. Interestingly, sciatic electrophysiological and skin intra-epidermal nerve fiber density (IENFD) peripheral measurements were not affected by the *APOE* genotype or HFD, suggesting that the observed sensorimotor and cognitive phenotypes are related to central nervous system processes. Indeed, measurements of hippocampal insulin receptor and glycogen synthase kinase-3β (GSK-3β) activation revealed a pattern similar to that obtained in the behavioral measurements while Akt activation presented a dominant effect of diet. HFD manipulation induced genotype-independent hyperlipidation of apoE, and reduced levels of brain apoE in apoE3 mice, rendering them similar to apoE4 mice, whose brain apoE levels were not affected by the diet. No such effect was observed in the peripheral plasma levels of apoE, suggesting that the pathological effects of apoE4 under the control diet and apoE3 under HFD conditions are related to the decreased levels of brain apoE. Taken together, our data suggests that diabetic mechanisms play an important role in mediating the pathological effects of apoE4 and that consequently, diabetic-related therapy may be useful in treating apoE4 pathology in AD.

## 1. Introduction

### 1.1. Alzheimer’s Disease and Apolipoprotein ε4

Alzheimer’s disease (AD) is the most common form of dementia, an age-related neurodegenerative disorder affecting more than 50 million people worldwide [1,2]. It is clinically characterized by progressive deterioration of cognition, loss of insight, judgment, language, and changes in behavior and, in late stages, physical functioning [3]. Neuro-biochemical assessments of AD patients’ brains revealed selective impairments in the hippocampus and neocortex areas, with damage to mainly cholinergic neuronal populations [4,5]. Along with neuronal loss, AD pathology is characterized by the occurrence of senile plaques, which contain Aβ depositions, neurofibrillary tangles composed of hyper-phosphorylated tau, and extensive synaptic loss [6]. The sex-based prevalence of AD is well documented, with over 60% of AD patients being female [7]. An even greater sex difference has been found in the impact of AD pathology, with each additional unit of AD pathology found to be associated with a nearly 3-fold increase in the odds of clinical AD in men compared with a more than a 22-fold increase in the odds of clinical AD in women [8]. AD is manifested either in a familial or sporadic form; the familial form is characterized by mutations in the amyloid precursor protein (APP) and presenilin 1 and 2, resulting in elevated levels of Aβ deposits and senile plaques [9,10]. Studies of sporadic AD revealed allelic segregation of the apolipoprotein E (*APOE*) gene to families and individuals with a higher risk of late-onset sporadic AD [11,12,13]. There are three main alleles of the apolipoprotein E, termed apoE2, apoE3, and apoE4, of which the ε4 gene allele (apoE4) has been found to be the most prevalent genetic risk factor for AD [11,12,14]. The frequency of this allele in AD patients is over 50%, whereas in the general population its frequency is about 25% [15]. Each ε4 allele reduces the age of AD onset by 7 to 9 years per allele copy [12,16]. Evidence indicates that the apoE4 risk for AD is greater in women than in men [7]; a single copy of the ε4 allele in women is sufficient to increase the AD disease risk associated with two copies of ε4 in men [17]. Furthermore, it has been shown that carrying the apoE ε4 allele has a larger deleterious effect on neurodegeneration, synaptic plasticity, and adult neurogenesis, and on cognitive performance in females more than males [18].

Neuropathologically, apoE4 is associated with a wide range of brain-related phenotypes, including impaired neurite outgrowth, synaptogenesis, and plastic neuronal remodeling, as well as increased neurodegeneration [19,20,21]. As opposed to the effect of apoE4 on the CNS, there is a lack of understanding regarding its effect on the peripheral nervous system (PNS) [22,23]. This is despite the fact that apoE is expressed throughout the PNS, including at the neuromuscular junction (NMJ) [24,25], where expression levels dramatically increase in response to nerve crush injury and exposure to harmful environmental stimuli [26]. An association between the *APOE* genotype and disease outcome has been demonstrated for human patients with diabetic neuropathies [27,28], but very few have investigated the influence of the apoE isoform on the PNS in genetically modified animals [26]. In general, although many studies have been carried out regarding AD, the etiology and pathogenesis of the disease are not yet fully understood [29]; therefore, new links that may unravel the answers to these questions are necessary.

### 1.2. Alzheimer’s Disease and Type 2 Diabetes Miletus

Epidemiologic studies revealed that the risk of AD is increased by 50%–100% by type 2 diabetes miletus (T2DM) [30,31,32]. A recent population-based study found that the overall incidence densities of AD for diabetic men and women, respectively, were 0.82 and 1.15 per 1000 person-years, with an especially notable higher hazard ratio of AD in older diabetic women [33]. T2DM is a metabolic disorder of impaired glucose regulation, one of the most common chronic metabolic diseases, with more than 300 million patients worldwide. Clinically, T2DM is characterized by a slow decline in the functions of multiple body organs and systems, and impairments in cognitive functions [34]. Pathological characteristics include peripheral and central impaired insulin action, insulin secretory deficiency, and increased endogenous glucose production [35]. Diabetic peripheral neuropathy (DPN) is a common late complication of diabetes, defined as a symmetric length-dependent sensorimotor polyneuropathy attributable to metabolic and microvascular alterations as a result of chronic hyperglycemia exposure (diabetes) [36,37]. DPN is characterized by loss of small-fiber–mediated sensation, resulting in the loss of thermal and pain perception, and large-fiber impairment resulting in the loss of touch and vibration perception [37]. T2DM and AD have been shown to share many pathophysiological features [38], which include insulin resistance, disrupted glucose metabolism, peripheral oxidative and inflammatory stress, amyloid aggregation, neural atrophy, neurodegeneration, and cognitive decline [30]. Furthermore, key pathological changes that occur in an AD brain, such as amyloid aggregation, resemble those that occur in the pancreas and vasculature of diabetic patients [39,40]. These findings and more led to the hypothesis that impaired neuronal insulin action might be a unifying pathological mechanism in the development of both T2DM and AD [41]. Indeed, insulin resistance and impaired insulin signaling in the central nervous system (CNS) have been linked to the pathogenesis of AD, observations that resulted in terming AD “type 3 diabetes” [30,42,43].

The expression of insulin receptor (IR) units in the CNS displays a widespread but selective regional pattern, and they are found abundantly in brain areas involved in glucose and energy homeostasis as well as cognitive processes [41]. In rodents, one of the areas with the highest density of IRs is the hippocampus [44,45,46]. In the canonical insulin signaling pathway, insulin first binds to its receptor on the plasma membrane and induces signal transduction that includes activation/phosphorylation of Akt on residues Thr308 and Ser473 [35]. In turn, activated Akt inhibits glycogen synthase kinase-3β (GSK-3β) by inhibition/phosphorylation of Ser9 residues [47,48]. GSK-3β is an inhibitor of glycogen synthase (GS), and the Akt-driven inhibition of GSK-3β thus results in the promotion of glycogen synthesis and glucose absorption [35,49]. Additionally, GSK-3β has been found to be activated by auto-phosphorylation of Tyr216, which in AD can result in pathological and abnormal phosphorylation of tau [50]. In the brain, the insulin signaling pathway plays a significant role in neuronal health as well as synapse formation and maintenance. Significant impairments in this pathway have been documented in both postmortem analysis and animal models of AD [51]. Furthermore, insulin resistance may also contribute to cognitive deficits in AD, seeing that healthy brain insulin signaling has been found to be crucial for learning and memory [43,52].

### 1.3. ApoE4 and T2DM

Given the overlap of AD and T2DM, several anti-diabetic agents have been tested for treatment of AD, with many showing reduced benefits in apoE4 patients, such as intranasal insulin treatment and insulin sensitizer Rosiglitazone trials [53,54]. Although both apoE4 carriers and non-carriers with AD appear to have a brain insulin defect, the apoE4 carrier status appears to modulate the relationship between the peripheral and brain insulin metabolism [53]. These findings suggest an association between two major risk factors for AD, apoE4, and T2DM. The ε4 allele has been found to be an independent risk factor for T2DM [55] and associated with the development of T2DM [56]. Furthermore, the presence of apoE4 exacerbates AD neuropathology in the presence of T2DM [57]. For example, apoE4 enhances the differences between T2DM and non-T2DM subjects in the number of hippocampal and cortical neuritic plaques, neurofibrillary tangles, and the load of cerebral amyloid angiopathy [58]. It has also been shown that in apoE4 carriers, the degree of glucose dysregulation (evaluated by fasting blood glucose concentration and mean glycemic value, as measured by the HbA1c concentration [59]) correlates with reduced cortical thickness and that apoE4 carriers with T2DM demonstrate a level of cortical thinning comparable to that of preclinical AD [60]. Metabolic deficiencies, such as abnormally low rates of brain glucose metabolism, that are unrelated to regional amyloid burden [61] were observed in healthy apoE4-positive volunteers as young as their twenties [62]. Additionally, altered expression of insulin signaling parameters has been observed in apoE4 mouse and human brains [63,64]. However, despite the strong body of evidence linking T2DM and apoE4, the mechanism underlying the interaction between these two critical risk factors for AD is yet to be determined [54].

In the present study, we employed a dietary regimen of a high-fat diet (HFD), a well-established and robust animal model for studying impaired glucose tolerance and early T2DM in mice [65,66], compared to a regular diet, on an *APOE*-targeted replacement (TR) mouse model expressing either apoE3 or apoE4. Utilizing this model, we investigated the effects of the *APOE* genotype and HFD on glucose metabolism and insulin resistance, and the extent to which these are associated with apoE4-driven peripheral or central pathologies.

## 2. Results

### 2.1. Body Weight and Glucose Metabolism

**Figure 1:**
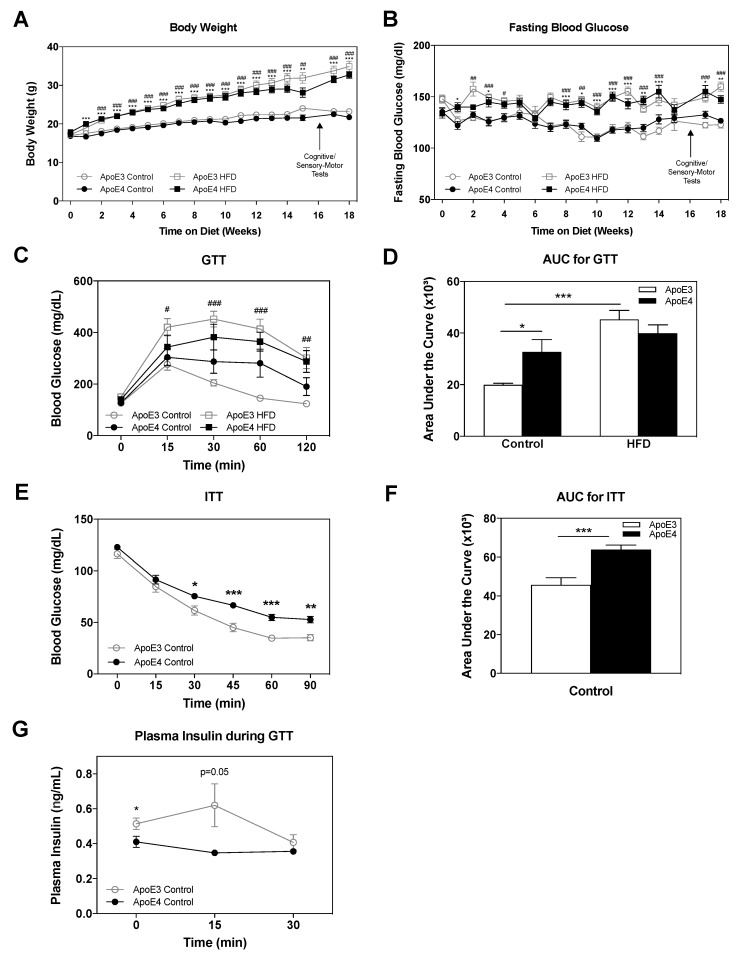
The effects of *APOE* genotype and high fat diet (HFD) on body weight, glucose metabolism, and insulin resistance. Following weaning, apoE4 and apoE3 mice were subjected to either a control or HFD for 18 weeks. (**A**) Body weight and (**B**) fasting blood glucose were monitored weekly following a 6-h fast as indicated. *n* = 17–18 mice/group. ApoE4 control and HFD comparisons are represented by *, apoE3 control and HFD comparisons are represented by #. (**C**) Glucose tolerance test (GTT). Blood glucose levels were measured at the indicated time points after an intraperitoneal injection of glucose. ApoE3 control and HFD comparisons are represented by #. *n* = 8 mice/group. (**D**) Quantitation of GTT results expressed as area under the curve. (**E**) Insulin tolerance test (ITT). Blood glucose levels were measured at the indicated time points following an intraperitoneal injection of insulin. *n* = 10 mice/group. (**F**) Quantitation of ITT results expressed as area under the curve. (**G**) Insulin levels in plasma at the indicated time points of GTT. *n* = 6–7. The areas under the curve of the GTT in (**D**) and ITT in (**F**) were calculated using the trapezoidal rule. Data were analyzed via two-way ANOVA, repeated measures when appropriate, with Bonferroni post-hoc comparisons; ITT results were analyzed via Student’s *t-*test. Data is presented as mean ± SEM, */# *p* < 0.05, ** /##*p* < 0.01 and ***/### *p* < 0.001.

Following weaning at 7 weeks of age, mice were subjected to either a HFD or regular chow diet for 18 weeks, as described in the materials and methods, utilizing *n* = 17–18 mice per group. Throughout these 18 weeks, body weight and fasting blood glucose levels were monitored once a week. The results thus obtained are presented in Figure 1A,B. As can be seen in Figure 1A, there was an age-dependent increase in the weight of apoE3 and apoE4 control diet mice, which was similar in both genotypes (23.23 ± 0.4 and 21.71 ± 0.4 at 18 weeks on diet; respectively). The HFD induced a marked and similar increase in body weight of apoE3 and apoE4 mice (34.89 ± 1.4 and 32.72 ± 0.9 at 18 weeks on diet; respectively). It is important to note that starting at week 15 on the HFD, apoE4 HFD-fed mice showed a trend of decreased weight gain in comparison to apoE3 HFD-fed mice. As can be seen in Figure 1B, the HFD also induced an increase of fasting blood glucose, which was similar between apoE3 and apoE4 mice, in accordance with the corresponding body weight measurements (160 ± 4.5 and 147.3 ± 3.6 at 18 weeks; respectively), whereas control diet apoE3 and apoE4 mice did not differ in fasting blood glucose (122.7 ± 3.2 and 126.7 ± 2.5 at 18 weeks; respectively).

The effects of the HFD and apoE genotype on glucose and insulin tolerance were next evaluated. Glucose tolerance was assessed via an intraperitoneally (IP) glucose tolerance test (GTT) as described in the materials and methods (*n* = 8 mice/group). As can be seen in Figure 1C, apoE4 control diet mice displayed delayed recovery compared to the corresponding apoE3 control diet mice, suggesting impaired glucose tolerance in apoE4 mice. HFD treatment resulted in increased glucose levels in both the apoE3 and apoE4 mice, indicating a pathological glucose tolerance. Areas under the curve (AUC) of GTT measurements were calculated using the trapezoidal rule [67], and the results are depicted in Figure 1D. Two-way ANOVA analysis revealed a significant effect for genotype (*** *p* < 0.0001) and interaction diet x genotype (*p* = 0.01), Bonferroni post-hoc comparisons showed significantly increased glucose levels in apoE4 mice relative to apoE3 mice (33 ± 5 and 20 ± 6, respectively; * *p* < 0.05), while apoE3 and apoE4 HFD mice’s AUC was elevated in a similar manner (45 ± 3 and 40 ± 3, respectively). ApoE3 control diet mice displayed significantly lower (*p* < 0.001) glucose levels relative to apoE3 HFD mice.

Insulin tolerance was assessed via the IP-insulin tolerance test (ITT) (*n* = 10 mice/group). In view of the findings that the differences in glucose tolerance between apoE3 and apoE4 mice were more pronounced in control diet mice, these experiments were performed on mice maintained on a control diet. As can be seen, in Figure 1E, the injection of insulin in both groups resulted in decreased levels of blood glucose. This decrease was more gradual and less pronounced in apoE4 mice. Quantitation of these results in terms of total glucose levels by measurements of AUC revealed increased levels of glucose in apoE4 compared to apoE3 mice. Accordingly*,* the Student’s *t-*test for AUC of the ITT results revealed a significant effect (*** *p* < 0.001) for the comparison of apoE3 and apoE4 control diet mice (46 ± 4 and 64 ± 2, respectively) (Figure 1F). These results suggest that apoE4 is associated with increased insulin resistance relative to apoE3 mice.

Insulin levels in the plasma were assessed at baseline, 15 and 30 min following glucose injection in the GTT as described in the materials and methods, and the results are depicted in Figure 1G. As can be seen, the levels of insulin in the plasma are elevated in apoE3 mice relative to apoE4 mice at baseline (0.514 ± 0.03 and 0.41 ± 0.03, respectively; * *p* = 0.04) and 15 min following the glucose injection (0.62 ± 0.12 and 0.35 ± 0.01, respectively; *p* = 0.05), suggesting that there is a deficit in the ability of the apoE4 mice to secrete insulin in response to elevated blood glucose levels. Together with the GTT and ITT results, the results obtained suggest that the pathological effects of apoE4 are mediated by two complementary mechanisms: reduced ability to secrete insulin and increased insulin resistance.

### 2.2. Cognitive and Sensorimotor Tests

The effects of the HFD and *APOE* genotype on cognitive and sensorimotor parameters were next examined.

**Figure 2:**
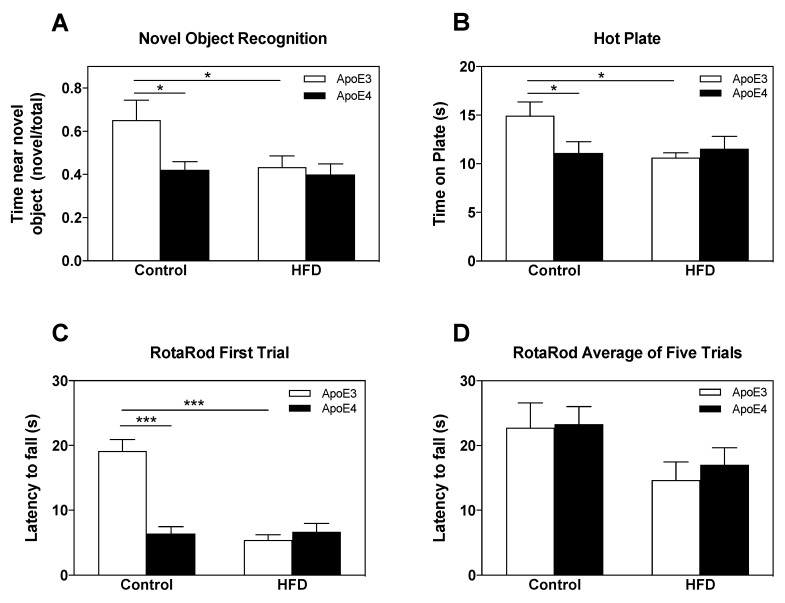
The effects of the *APOE* genotype and high fat diet (HFD) on short-term memory, nociception, and motor skills. ApoE4 and apoE3 mice, maintained on either a control diet or HFD, were subjected to cognitive and sensorimotor tests as described in the materials and methods. (**A**) Novel object recognition test (NOR). The results obtained are depicted as the fraction of the time spent near the novel object from the total time spent near both familiar and novel objects following 2.5 h from initial exposure to identical objects. *n* = 10 mice/group. (**B**) Hot plate test. Reactivity to thermal pain was measured by latency to nociceptive behavior on the hot plate (52 °C). *n* = 10 mice/group. (**C)** (**D**) Rotarod test. Motor coordination and learning were evaluated by measurements of latency to fall during the accelerating rotarod apparatus (5 to 50 RPM in 300 s). (**C**) Average time spent on the rotarod during the first round. (**D**) Average time spent on the rotarod during five consecutive trials. *n* = 10 mice/group. Data were analyzed via two-way ANOVA with Bonferroni post-hoc comparisons and the results were normalized relative to apoE3 control diet mice. Data are presented as mean±SEM. * *p* < 0.05 and ***p < 0.001.

Short-term memory was assessed via the novel object recognition (NOR) paradigm, in which the mouse’s tendency to approach a novel object was measured (*n* = 10 mice/group). Mice were exposed to a novel object 2.5 h following initial exposure to two similar objects (in which no preferences existed in either of the groups). As can be seen in Figure 2A, control diet apoE4 mice displayed no preference and visited the new and old objects similarly, whereas control diet apoE3 mice spent more time near the novel object, suggesting an intact short-term memory of the old object. This difference between the control diet apoE3 and apoE4 mice was abolished by the HFD, seeing that under these conditions, both apoE3 and apoE4 mice displayed no preference for the novel object. Statistical analysis via two-way ANOVA revealed a significant effect for genotype (*p* = 0.034). Further Bonferroni post-hoc comparisons revealed that the decreased preference of control apoE4 mice to the new object relative to that of the control apoE3 mice was significant (0.42 ± 0.04 and 0.65 ± 0.09, respectively; * p < *0.05*), as was the effect of the HFD on apoE3 mice relative to apoE3 control diet mice as seen by a lack of preference of apoE3 HFD mice (0.43 ± 0.05; * p < *0.05*). The HFD did not affect the performance of the apoE4 mice.

The effect of the *APOE* genotype and HFD on nociception was assessed by testing the latency of reactivity to thermal pain in the hot plate test (52 °C) (*n* = 10 mice/group). As can be seen, apoE4 control diet mice displayed hypersensitivity compared to apoE3 control diet mice while the HFD manipulation elevated sensitivity in apoE3 mice, rendering them similar to apoE4 mice. Analysis of the results via two-way ANOVA revealed a significant effect for the interaction between genotype x diet (*p* < 0.05). As can be seen in Figure 2B, apoE4 control diet mice showed statistically increased heat sensitivity relative to apoE3 control diet mice (11.11 ± 1.2 and 14.94 ± 1.4, respectively; * p < 0.05) while, similar to the effect seen in the NOR paradigm, the HFD abolished these differences and increased the thermal sensitivity of the apoE3 mice but did not affect the apoE4 mice (10.62 ± 0.5 and 11.54 ± 1.28, respectively).

Motor coordination and learning were tested using the rotarod apparatus, in which the amount of time mice spent on an accelerating rod was documented. The results thus obtained during the first trial are presented in Figure 2C (*n* = 10 mice/group). As can be seen, a similar pattern to the results of the NOR and hot plate tests was observed, where apoE4 mice displayed impaired motor skills relative to apoE3 mice in the control diet, while the HFD impaired motor skills in apoE3 mice without affecting apoE4 mice. Two-way ANOVA revealed a significant effect (*p* < 0.0001) for the interaction between genotype x diet. Bonferroni post-hoc comparisons revealed that apoE4 control diet mice displayed significantly decreased latency to fall relative to the apoE3 control diet mice (6.44 ± 1 and 19.13 ± 2, respectively; *** p < 0.0001). The HFD decreased the performance of apoE3 mice while no effect was seen in apoE4 HFD mice (5.4 ± 0.8 and 6.68 ± 1.3, respectively). This effect, however, was diminished over the four subsequent trials (Figure 1D), suggesting an effect on basal motor coordination, which was abolished in time by learning. The average performance of both genotypes throughout the five trials was similar in the control diet and tended to be decreased by the HFD. Indeed, statistical analysis revealed a significant effect (*p* < 0.05) for diet, whereas post-hoc comparisons showed no significant differences between groups.

Taken together with the results of the NOR, the hot plate and first trial of the rotarod test revealed a similar pattern in which apoE4 mice were impaired relative to apoE3 control diet mice, and the HFD damaged the performance of the apoE3 mice, rendering them similar to apoE4 mice, which were not affected by the HFD.

### 2.3. PNS Skin Intra-Epidermal Nerve Fiber Density (IENFD) and Sciatic Nerve Electrophysiological Measurements

**Figure 3:**
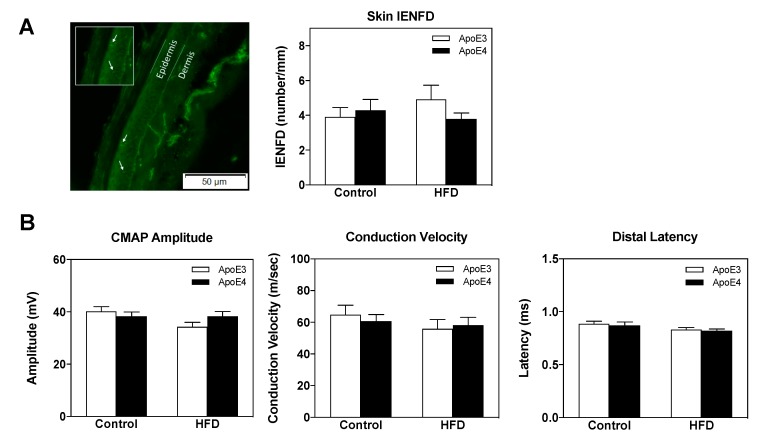
Skin intra-epidermal nerve fiber density (IENFD) and electrophysiological findings in apoE4 and apoE3 mice maintained on a control or high fat diet (HFD). (**A**) Skin IENFD measurements were conducted on footpad tissue, which was reacted with anit-PGP9.5 Ab as described in the materials and methods. A representative image of apoE3 control diet mice is depicted on the left panel, green fluorescence corresponds to PGP9.5 and white arrows correspond to intra-epidermal nerves. Quantitation of nerve fiber density per mm is presented in the right panel. *n* = 8–10 mice/group. (**B**) Electrophysiological measurements were conducted on the sciatic nerve as indicated in the materials and methods. Proximal compound muscle action potential (CMAP) amplitudes are depicted in the left panel. Motor nerve conduction velocity (MNCV) is presented in the middle panel. Distal latency was measured and MNCV was calculated as per the measured distance between stimulating cathodes. Distal latency is presented in the right panel. *n* = 10 mice/group. Data were analyzed via two-way ANOVA with Bonferroni post-hoc comparisons and the results were normalized relative to apoE3 control diet mice. Results are mean ± SEM.

The extent to which the observed effects of the *APOE* genotype and HFD on cognitive and sensorimotor parameters correlate with peripheral neuropathy was next assessed via skin IENFD and sciatic nerve electrophysiological measurements. Accordingly, we assessed footpad intra-epidermal nerve fiber density (Figure 3A) following 18 weeks on either a control or HFD (*n* = 8–10 mice/group). There were no significant effects of either the *APOE* genotype or diet on these parameters. Compound muscle action potential (CMAP), nerve conduction velocities, and distal latencies of the sciatic nerve were next examined (Figure 3B) (*n* = 10 mice/group). Similar to the skin IENFD results, these measurements revealed no effect of either the *APOE* genotype or HFD on the said parameters. Taken together, these findings suggest that the demonstrated behavioral effects of apoE4 and HFD are not associated with peripheral small fiber neuropathy or sciatic nerve electrophysiological pathologies.

### 2.4. CNS Insulin Signaling

We next focused on the effects of the HFD and *APOE* genotype on insulin signaling parameters in the hippocampus. This was performed by utilizing immunoblots of the insulin receptor (IR), and the subsequent signaling molecules Akt and GSK-3β.

**Figure 4:**
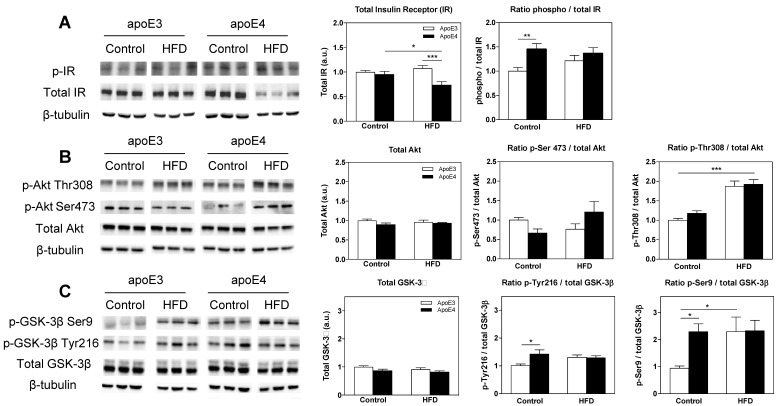
The effects of the *APOE* genotype and high fat diet (HFD) on insulin signaling parameters in the hippocampus. Hippocampal homogenates of apoE3 and apoE4 mice subjected to a control diet or HFD were blotted and reacted with indicated antibodies as described in the materials and methods. Representative immunoblots of three mice per group are presented on the left panel along with the β-tubulin loading control. Quantitation of the bands is presented in the right panels, such that the total levels of the parameter are presented in the left graphs and activated levels in the right graphs as presented by the ratio of the phosphorylated molecule to its total level. (**A**) Total and phosphorylated insulin receptor (IR) levels. *n* = 15–22 mice/group. (**B**) Total Akt, p-Akt Thr308, and p-Akt Ser273. *n* = 6–11 mice/group. (**C**) Total glycogen synthase kinase-3β (GSK-3β), p-GSK-3β Tyr216, and p-GSK-3β Ser9. *n* = 11–17 mice/group. ApoE3 mice are depicted in white bars and corresponding apoE4 mice are depicted in black bars. β-tubulin was used as a loading reference, and the results presented were normalized relative to the apoE3 control diet mice. Data were analyzed via two-way ANOVA with Bonferroni post-hoc comparisons, and results are mean ± SEM; * *p* < 0.05, **p < 0.01, and *** *p* < 0.001.

The results of the total and activated IR levels are presented in Figure 4A. As can be seen, apoE3 and apoE4 mice displayed similar levels of total IR under basal control diet conditions while the HFD decreased levels or IR in apoE4. Two-way ANOVA of the total IR levels revealed a significant effect for the interaction between genotype x diet (*p* = 0.009). Post-hoc comparisons showed that the total IR levels were similar in the control diet groups and that the HFD significantly reduced levels in apoE4 mice relative to apoE4 control diet mice (0.74 ± 0.1 and 0.96 ± 0.1, respectively; * *p < 0.05)*. Furthermore, apoE3 HFD mice revealed significantly higher IR levels relative to apoE4 HFD (1.1 ± 0.1 and 0.74 ± 0.1, respectively; *** p < 0.001).

Measurements of the activated IR, as presented by the ratio of phosphorylated IR to its total level, revealed elevated levels in apoE4 mice relative to apoE3 mice in the control diet groups while the HFD abolished these differences, rendering apoE3 mice similar to apoE4 mice. Two-way ANOVA revealed a significant effect for genotype (*p* = 0.0075), and Bonferroni post-hoc comparisons showed that apoE4 control diet mice displayed significantly higher IR activity relative to apoE3 control diet mice (1.45 ± 0.1 and 1 ± 0.08, respectively; ** *p* < 0.01). The results of all IR immunoblots represent two cohorts, total *n* = 15–22 mice/group.

Akt is activated by insulin-mediated phosphorylation of Thr308 and Ser473, with Ser473 phosphorylation also driven by mechanistic target of rapamycin complex 2 (mTORC2) [68]. The effects of the *APOE* genotype and HFD on total levels and said phosphorylation states of Akt are depicted in Figure 4B. As can be seen in the left graph, the total levels of Akt were not changed by either the *APOE* genotype or diet, which is consistent with previous observations [69] (Figure 4B). In contrast, the extent of Akt activation, as measured by the ratio of p-Akt Ser473 to total Akt, revealed a significant effect for interaction between genotype x diet (*p* = 0.024). In accordance with previous findings [69], apoE4 control diet mice displayed a trend of decreased Ser473-mediated activation of Akt relative to apoE3 control diet mice (0.664 ± 0.1 and 1 ± 0.1, respectively). Interestingly, this trend was turned around in the HFD mice, where apoE4 mice showed a trend of increased activation relative to apoE3 mice (1.21 ± 0.3 and 0.76 ± 0.14, respectively). The corresponding analysis of the extent of Akt activation, as measured by the ratio of *p*-Akt Thr308 to total Akt, revealed a significant effect for diet (*p* < 0.0001). Thr308-mediated Akt activation revealed a small increase in apoE4 control diet mice relative to apoE3 control diet mice, and HFD resulted in a marked similar increase in both genotypes. Statistical analysis revealed significantly higher levels of Thr308 activation in the apoE4 HFD mice relative to apoE4 control diet mice (1.92 ± 0.1 and 1.18 ± 0.1, respectively; *** p < 0.001), as in apoE3 HFD compared to apoE3 control diet mice (1.87 ± 0.1 and 1 ± 0.05; *** p < 0.001, respectively). *n* = 6–11 mice/group.

GSK-3β is generally active in the absence of exogenous signals and is thus acutely inactivated by Akt via inhibitory phosphorylation on Ser9. Activating autophosphorylation of Tyr216 has been shown to further regulate the kinase’s activity. The results of the GSK-3β immunoblot measurements are depicted in Figure 4C. As previously shown [69], the levels of total GSK-3β were similar in apoE3 and apoE4 control diet mice, and no significant differences between genotypes were found following the HFD (Figure 4B). In terms of activation-phosphorylation of GSK-3β on Tyr216, apoE4 control diet mice showed significantly higher activation than apoE3 control diet mice (1.42 ± 0.2 and 1 ± 0.1, respectively; * p < 0.05) as previously shown [69]. Interestingly, this effect was abolished by the HFD. Two-way ANOVA revealed a significant effect for the interaction between genotype x diet (*p* = 0.0425). The activity of phosphorylated Ser9, which is a marker of inhibited GSK-3β, is presented in the right panel in Figure 4C. As can be seen, the inhibited activity of GSK-3β via Ser9 phosphorylation was higher in apoE4 control mice relative to apoE3 control mice, and HFD administration resulted in a marked increase in apoE3 mice, rendering them equal to apoE4 mice, which were not affected by the HFD. Two-way ANOVA statistical analysis revealed significantly higher levels of activation in apoE4 control diet mice relative to apoE3 control diet mice (2.23 ± 0.3 and 1 ± 0.1, respectively; * p < 0.05) while the HFD abolished these differences and significantly increased the levels of Ser9-mediated GSK-3β inhibition in apoE3 mice to the levels of the apoE4 control diet mice but did not affect levels of activity in apoE4 mice. Post-hoc comparisons revealed apoE3 control diet mice displayed significantly lower levels relative to apoE3 HFD (1 ± 0.1 and 2.3 ± 0.5, respectively; * p < 0.05) mice. The results of all GSK-3β-related parameters represent two cohorts, total *n* = 11–17 mice/group.

Taken together, the insulin signaling measurements demonstrate that apoE4 induces insulin and glucose metabolism-related deficits and that these effects are associated with marked behavioral effects.

### 2.5. ApoE Lipidation and Levels in the Brain and Plasma

**Figure 5:**
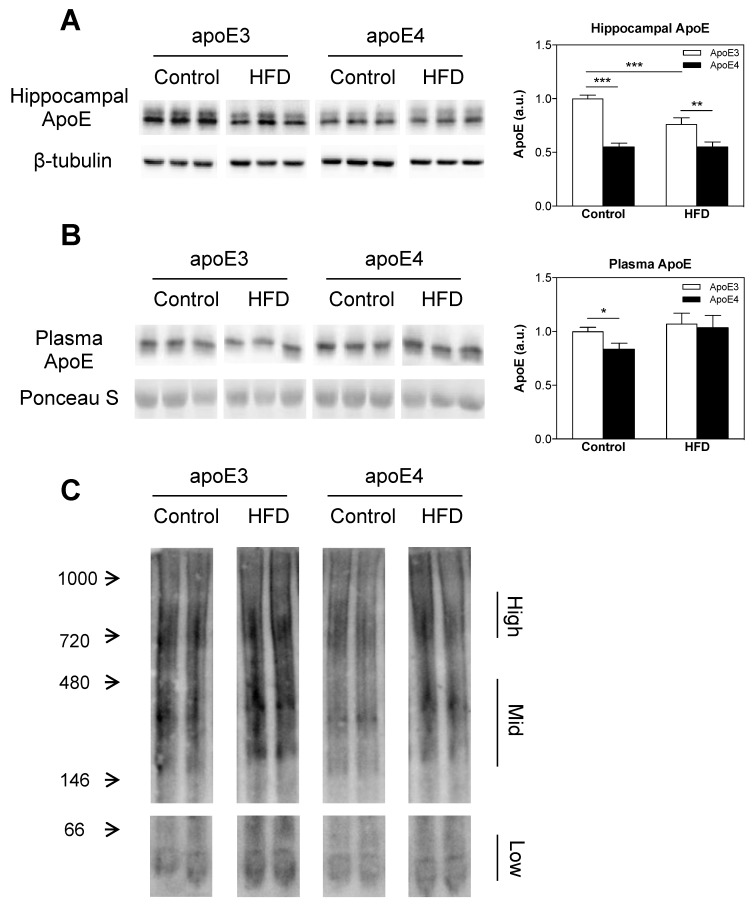
The effects of the *APOE* genotype and high fat diet (HFD) on apoE lipidation and levels in the hippocampus and plasma. Hippocampal homogenates (**A**) and plasma samples (**B**) of apoE3 and apoE4 control or HFD mice were blotted and reacted with anti-apoE Ab as described in the materials and methods. Representative immunoblots of three mice per group are presented on the left panel along with the loading control. Quantitation of the intensities of the apoE bands is presented in the right panel. (**A**) Hippocampal apoE levels. *n* = 15–17 mice/group. (**B**) Plasma apoE levels. *n* = 8–10 mice/group. (**C**) ApoE lipidation. Control and HFD apoE3 and apoE4 hippocampal homogenates were subjected to a blue native gel and stained with anti-apoE Ab as described in the materials and methods. Representative immunoblots of two mice per group are presented on the left panel. *n* = 3 lanes per group, each of the lanes represents a pull of 3 separate mice of the same group. Results were normalized relative to control diet apoE3 mice. Data were analyzed via two-way ANOVA with Bonferroni post-hoc comparisons and results are mean ± SEM; * *p* < 0.05, ** *p* < 0.01, and *** *p* < 0.001.

The effects of the *APOE* genotype and HFD on apoE levels in the brain and plasma were assessed via immunoblots of hippocampal homogenates and plasma extracts from apoE3 and apoE4 control and HFD-fed mice. In accordance with previous findings [70], the total hippocampal apoE levels were significantly lower in apoE4 control diet mice relative to apoE3 control diet mice (0.55 ± 0.03 and 1 ± 0.03, respectively; *** *p* < 0.001). Interestingly, the HFD significantly decreased (*** *p* *<* 0.001) the levels of hippocampal apoE in apoE3 HFD mice relative to apoE3 control diet mice, rendering them closer to the levels of apoE4 control mice without affecting apoE4 mice (Figure 5A). Still, hippocampal apoE levels in apoE4 HFD mice remained significantly lower (** *p* < 0.01) relative to apoE3 HFD mice (0.55 ± 0.04 and 0.763 ± 0.06, respectively). Two-way ANOVA revealed a significant effect (*p* < 0.0001) for genotype and a significant effect (*p* < 0.01) for diet and the interaction between genotype x diet. The results of the hippocampal apoE immunoblots represent two cohorts, total *n* = 15–17 mice/group.

Plasma apoE levels were measured next. This revealed that similar to the brain phenotype and as previously described [71], the levels of plasma apoE in control apoE4 mice were significantly lower than those of the corresponding apoE3 mice plasma apoE relative to the apoE3 control diet (0.84 ± 0.05 and 1 ± 0.04, respectively; * *p* < 0.05), as revealed by post-hoc comparisons of two-way ANOVA. In contrast, there was no difference in the plasma levels of HFD-fed apoE4 and apoE3 mice, which was due to a small increase in the levels of plasma apoE in apoE4 mice. The results of the plasma apoE immunoblots represent two cohorts, total *n* = 8–10 mice/group.

The effects of the *APOE* genotype and HFD on apoE lipidation in the brain were assessed via blue native gel, and the results can be seen in Figure 5C, *n* = 3 lanes per group, where each lane represents a pull of three mice from the same group. In accordance with previous findings [70,72], under control diet conditions, apoE4 mice displayed hypolipidation of apoE relative to apoE3 mice. Importantly, the extent of lipidation of apoE in apoE3 and apoE4 mice was increased in mice exposed to the HFD, rendering the lipidation of apoE4 HFD mice similar to that of the apoE3 control diet mice.

Taken together, these observed results show that under normal diet conditions, the levels of apoE in apoE3 mice are significantly higher than those of apoE4 in the brain and plasma, an effect that correlates with hypolipidation of apoE in apoE4 mice. The HFD increases apoE lipidation in both apoE3 and apoE4 mice, and reduces brain apoE levels in apoE3 mice without further affecting apoE4 mice, but has no significant effect on the levels of apoE in the plasma of neither apoE3 nor apoE4 mice.

## 3. Discussion

The present study examined the effects of the *APOE* genotype and HFD on glucose metabolism and insulin resistance, and the extent to which they are associated with peripheral and central pathologies. The results thus obtained revealed that under basal conditions, apoE4 mice display impaired glucose tolerance relative to apoE3 mice while the HFD impairs apoE3 mice without significantly affecting apoE4 mice. Complementary measurements of insulin resistance and insulin levels revealed that the impairment of glucose tolerance in apoE4 mice maintained on a control diet is parallel to impairments in insulin tolerance and insulin secretion. Measurements of weight and fasting blood glucose revealed an effect of diet and not genotype, where the HFD increased body weight and fasting blood glucose in a time-dependent manner.

Behavioral cognitive and sensorimotor measurements revealed a similar pattern to that obtained in the glucose tolerance measurements, in which under basal conditions, apoE4 mice are impaired relative to apoE3 mice, and the HFD induces pathological behavioral consequences in apoE3 mice, rendering them similar to apoE4 mice whose behavior was not affected by HFD. Diabetic neuropathy impairments in thermal perception are associated with small-fiber-mediated sensation, and pathologies in touch perception and motor coordination are linked to large-fiber neuropathies [36,37,73]. Therefore, we next examined the extent to which the sensorimotor profile of the mice is affected by the *APOE* genotype and diet effects on small and large fibers utilizing skin IENFD for the first [74] and sciatic electrophysiological measurements [75] for the latter. These experiments revealed no significant effects of either the *APOE* genotype or diet on these peripheral parameters, suggesting that the observations recorded in the behavioral and sensorimotor paradigms, much like the observed cognitive phenotypes, are driven primarily by the CNS and not by the PNS. This assertion is supported by the finding that central insulin signaling-related parameters, particularly the activation of IR and GSK-3β, are affected by the apoE genotype and diet in a pattern similar to that of the glucose tolerance and cognitive and sensorimotor results. Interestingly, the associated Akt activity, which was not majorly affected by the *APOE* genotype, was increased similarly by the HFD in both genotypes, suggesting that the coupling between Akt and GSK-3β in the hippocampus is complex and governed by more than one mechanism. Further evidence supporting CNS processes as a driving force for the observed behavioral effects of the *APOE* genotype and diet was obtained by measurements of the apoE levels and lipidation. As previously shown, in both the brain and plasma, the levels of apoE in apoE4 mice are significantly lower than in apoE3 mice under basal conditions, effects that are accompanied by hypolipidation of brain apoE in apoE4 mice relative to apoE3 mice [71,72,76]. Interestingly, brain apoE levels were sensitive to both the *APOE* genotype and diet, seeing that the HFD reduced apoE levels in apoE3 mice, rendering them similar to apoE4 mice, which were not affected by the diet. No such effect was observed in the peripheral plasma apoE measurements. Furthermore, subjection to the HFD resulted in hyperlipidation of apoE, independent of genotype.

The current findings that apoE4 mice display impaired glucose and insulin tolerance as well as reduced insulin levels following elevated blood glucose, relative to apoE3 mice under control diet conditions, suggest that the pathological effects of apoE4 are mediated by two complementary mechanisms: Reduced ability to secrete insulin and increased insulin resistance. These observations are in line with a substantial body of literature showing that apoE4 is associated with various impairments in CNS metabolism, including decreased cerebral glucose uptake as well as reductions in cerebral glucose utilization observed in normal apoE4 individuals as young as their 20–30s [62]. Furthermore, the clinical finding that apoE4 carriers, unlike non-carriers, do not cognitively benefit from intranasal insulin administration is suggestive of the fact that they have increased insulin resistance [53]. The elevation of fasting blood glucose and weight in mice of both genotypes subjected to the HFD is also compatible with the literature [18,54] while in the present experiment weight gain tends to be less elevated in apoE4 mice in the final weeks of the HFD, an effect that was more dramatic in other studies [54,77,78]. In accordance with previous findings [79], this effect appears to increase with age, which may be why only a small difference was observed in our study. These findings together suggest that apoE4 carriage can in itself be viewed as a form of cerebral metabolic dysfunction [80].

The current finding that apoE4 carriers are impaired relative to apoE3 carriers in cognitive parameters is consistent with previous studies in mice [81,82,83] and humans [84,85,86]. The fact that the HFD impaired apoE3 mice is consistent with many studies that have shown that WT mice are impaired following the HFD in cognitive [87,88], sensory [89], and motor paradigms [89]. The cognitive performance of apoE4 mice on HFDs has shown mixed results, with either increased deficits in spatial memory [54] or no cognitive differences [18], while the observation of the current study, revealing that apoE4 mice are not affected by the HFD in these parameters, may be due to the strong pathological phenotype in this model of apoE4 control diet mice, generating a floor effect in apoE4 HFD mice.

In the present study, we observed no differences in the peripheral phenotypes measured, between young apoE3 and apoE4-TR mice in sciatic electrophysiological and footpad skin IENFD measurements, which assess neuropathic impairments in large and small fibers, respectively. Although studies focusing on apoE isoform-specific alterations in the PNS are scarce, these observations are in line with previous findings [26], which reported no differences in the function or development of uninjured mouse PNS following the expression of human apoE3 or apoE4. Specifically, Comley et al. [26] reported that electrophysiological measurements from apoE4 mice suggested normal neuromuscular synaptic function, without qualitative or quantitative differences in the morphology of the sciatic nerve or neuromuscular junctions between apoE4 and apoE3 mice. It is important to note that evidence of peripheral nerve variances between apoE3 and apoE4 exists, as Comley et al. [26] revealed that apoE4 expression disrupts peripheral nerve regeneration and subsequent neuromuscular junction re-innervation following nerve injury compared with apoE3.

Several studies have also studied the effects of apoE4 with or without HFD manipulation on insulin signaling, directly implicating apoE4 in pathways of insulin signaling [80]. For example, in both human *APOE*-TR mice and postmortem human brain tissue, apoE4 was found to reduce the expression of insulin signaling proteins [64,90], and apoE4 expression in mice exaggerated impairments in insulin signaling [80,83,91]. Traversy et al. [92] observed no difference between apoE3-TR and apoE4-TR mice in the levels of IR in brain capillaries while Chan et al. [76] reported more IR was immunoprecipitated with apoE3 than apoE4 in human postmortem frontal cortex AD samples. Q.R. Ong et al. [64] reported a reduction of Akt phosphorylation at Thr308 at 32 weeks and at Ser473 at 72 weeks of age in apoE4-TR mice as compared to apoE3-TR mice. Chan et al. [93] reported that in 26-week old mice, there was no difference in the expression and phosphorylation of insulin signaling proteins among APP, *APOE3*x*APP*, and *APOE4*x*APP* mouse brains while when the mice aged to 78 weeks, these proteins were markedly reduced in *APP* and *APOE4*x*APP* mouse brains.

The effects of the *APOE* genotype and diet on *APOE*-TR mice have recently been reported by Zhao et al. [48]. In line with the PNS observations reported in the current study, Zhao et al. [48] revealed no significant differences between apoE3-TR and apoE4-TR control diet mice in skeletal muscle and liver p-Akt (Ser473) and p-GSK3β (Ser9), indicating that basal peripheral insulin signaling remains intact in apoE4-TR mice. Regarding CNS insulin signaling, Zhao et al. [48] observed a marked age-dependent decrease apparent at 24 months of age but not at 12 or 4 months, accompanied by an exacerbation of these effects following subjection to an HFD at middle age in apoE4 mice. In comparison, the present study observed an apoE4-dependent effect on insulin signaling at 6 months of age, as shown by measurements of the total and activated levels of IR and GSK-3β activation via Tyr216 and Ser9 phosphorylation. Seeing that the mice used in this study have a background of α-synuclein −/−, previously shown to exacerbate apoE4 pathology at a younger age [70], the observed effects on the insulin cascade may be due to this model’s characteristic of a young age pathological phenotype. Another interesting variance in the results regards the effects of the HFD manipulation, where the present study found that the HFD impairs apoE3 mice, rendering them similar to apoE4 mice in various parameters; Zhao et al. [48] observed no effect on apoE3 mice following the HFD. In this context, it is interesting to note that many studies have shown that HFD-induced insulin resistance impairs control wildtype (WT) mice [91,94,95], meaning that the results revealed in the present study may be compatible with effects of this pattern. Although some specifics of the Zhao et al. study [48] and the current one differ, the general concept that CNS insulin signaling is affected by apoE4 while peripheral parameters are not seems to be consistent between the two. Overall, the mixed findings presented in the field of insulin signaling parameters may be due to the high sensitivity of signaling parameters to age [96], gender [97], and brain area [98,99].

Regarding the mechanism underlying the observed pathological effects of apoE4, we suggest that these may be related either to decreased levels of apoE or biochemical changes in this molecule following hypolipidation of apoE4. The proposed apoE-level hypothesis is based on the observations that the impairments of the apoE3 mice following exposure to the HFD are associated with a parallel decrease in the levels of hippocampal apoE in apoE3 mice, rendering them similar to that of apoE4 mice, and that these impairments are similar to those of apoE4 mice maintained on a control diet. However, since the HFD treatment also increases the lipidation of apoE in apoE4 and apoE3 mice, it is possible that this hyperlipidation, which is presumably enriched with the diet lipids, could negatively affect apoE3 activity and impair functions, such as cholesterol transport. Accordingly, both hypolipidation of apoE as observed in apoE4 mice under control diet conditions and hyperlipidation of apoE, such as that observed in the apoE3 mice following HFD treatment, impair the function of apoE. Importantly, the finding that the effects of the apoE genotype are more pronounced in apoE levels in the brain relative to those observed in the periphery supports the hypothesis that the presently observed effects are triggered by decreased levels of apoE.

In conclusion, the present findings that apoE4 stimulates diabetic-related effects, such as an increase in glucose tolerance and insulin resistance and a decrease in insulin secretion, which are associated with further downstream distinct brain pathologies, and that apoE3 under pro-diabetic conditions of the HFD induces similar pathological changes, suggest that diabetic mechanisms play an important role in mediating the effects of apoE4 on brain pathology. This implies that diabetic-related therapy may be helpful in counteracting the effects of apoE4 in AD and that anti-apoE4-related therapies [72,100] may be beneficial in blocking the neuropathological effects of T2DM in apoE4 carriers.

## 4. Materials and Methods

### 4.1. Mice

Endogenous mouse *APOE* was replaced by either human *APOE3* or *APOE4*, in order to create *APOE*-TR mice by gene targeting as previously described [101]. These mice were purchased from Taconic (Germantown, NY, USA), and were backcrossed at Taconic for eight generations after their preparation. To minimize possible genetic drifting between the apoE4 and apoE3 mice, which were offspring of the homozygous apoE4 and apoE3 mice generated by Taconic in 2001, they were further crossed by us with Harlan C57Bl/6JOlaHsd mice, which unlike the standard Jackson laboratory C57Bl/6J ApoEtm1.1(APOE∗4)Adpmc mice (Jackson Laboratories, Bar Harbor, ME, USA) were α-syn−/−. The resulting mice were then crossbred to yield apoE4 and apoE3 homozygous mice on an α-syn−/− background. Accordingly, the present experiments were performed with apoE3 and apoE4 homozygous mice on an α-synuclein −/− background, in which we have previously shown that the apoE4 phenotype is more pronounced than in α-synuclein +/+ expressing mice [70]. These mice are referred to herein as apoE3 and apoE4 mice, respectively. The *APOE* genotype of the mice was confirmed by PCR analysis (as can be seen in supplementary materials), as previously described [102]. All experiments were approved by the Tel-Aviv University Animal Care Committee, approval number 04-17-058 (date of approval expiration: 07/11/2021). Every effort was made to reduce animal stress and to minimize animal usage.

In the current study, we chose to focus on female mice, seeing that females are more susceptible to AD than males, and the interaction between gender and apoE4 has been demonstrated in numerous animal and human studies, resulting in the finding that apoE4-related pathology is more pronounced in females [18,69]. Furthermore, recent findings in our lab showed that apoE4-TR female mice have a more robust pathological phenotype than the corresponding male mice [70]. The results presented correspond to 2 female cohorts, consisting of 4 groups: 2 genotypes (apoE3 or apoE4) X 2 diets (control or HFD). The first cohort contained 10 mice/group and the second cohort contained 7–8 mice/group.

### 4.2. Diets

After weaning, at 7 weeks of age, the mice were randomized by body weight and assigned to either a standard rodent chow diet (Teklad 2018, Envigo, Huntingdon, Cambridgeshire, UK) or an HFD (60% kcal, D12492, Research Diets) consumed ad libitum. Weight and blood glucose were measured weekly following a 6-h fast, as described in the following sections. According to previous studies [103], diabetic symptoms appear in C57BL/6 mice after a minimum of 11 weeks on the HFD. Therefore, starting at 15 weeks on the diet, the mice underwent cognitive tests and either metabolic or motor and sensory tests. At the age of 6 months, the mice were anesthetized with ketamine and xylazine, after which blood samples were collected from the vena cava and mice were perfused transcardially with phosphate-buffered saline (PBS). Their brains were then removed, and the hippocampi were further processed for biochemical analysis.

### 4.3. Metabolic Tests

The mice were subjected to the GTT 15 weeks following diet initiation, and in the following week to the ITT.

#### 4.3.1. GTT

Glucose tolerance measurements were performed as previously described [104,105]. In brief, following a 6-h fast, mice were weighed, and baseline blood glucose levels were measured using an Accu-Chek^®^ Performa glucometer (Roche, Welwyn Garden City, Hertfordshire, UK). Next, 2 g/kg body weight of d-glucose (Merck, Darmstadt, Germany) were IP injected (e.g., 250 µL of 20% glucose solution for a mouse weighing 25 g), and glucose levels from tail blood samples were collected and measured 15, 30, 60, and 120 min post-injection. To measure insulin levels, blood samples were collected at baseline as well as 15 and 30 min post-injection and plasma was isolated. Plasma insulin was measured using a Mouse Insulin detection ELISA kit (Mercodia, Uppsala, Sweden).

#### 4.3.2. ITT

Insulin tolerance measurements were performed as previously described [104,105]. In brief, mice were fasted for 6 h, after which they were weighed and baseline blood glucose levels were measured, as described above. This was followed by an intraperitoneal injection of 1.5 U/kg body weight of insulin (Humulin^®^-R-100, Eli Lilly, Indianapolis, IN, USA) (e.g., 375 µL of 0.1 U/mL insulin solution for a mouse weighing 25 g). Blood glucose was measured at 15, 30, 45, 60, and 90 min following injection.

### 4.4. Behavioral Tests

The tests were initiated 15 weeks post the beginning of the diet administration. The mice were first subjected to the NOR test. After a 1-week interval, they underwent the rotarod test and 3 days later the hot plate test.

#### 4.4.1. Short-Term Memory Measurements

This test was performed as described in [81,106] and is based on the natural tendency of rodents to investigate a novel object. For the habituation phase, the mice were first placed in an arena (50 × 50 cm with 40 cm high walls) in the absence of objects. After 24 h, the mice were placed back in the arena with two identical objects (A1 and A2, respectively) for a control test. Two and a half hours later, the mice were re-introduced to the arena in which one of the familiar objects was replaced by a novel one in order to test short-term memory (A1 and B, respectively). All objects presented similar textures, colors, and sizes but distinctive shapes. The behavior of the mice was then monitored using the EthoVision XT 13.0 program for 5 min, by the duration and number of visits that the mice paid to each of the objects. The results are presented as the ratio in the percent of the time spent near the novel object relative to the total time spent near both new and old objects, where values >0.5 are indicative of a preference for the new object.

#### 4.4.2. Motor Coordination and Learning Measurements

Motor performance and learning were tested using the rotarod paradigm (Rotor-Rod; San Diego Instruments, San Diego, CA, USA). Testing consisted of five trials in which each mouse was placed on the rod, which accelerated from 5 to 50 RPM over the trial time of 300 s. Trials were terminated when animals fell off the rod or the maximum time was reached. The rod was placed at a height of 45 cm, and mice fell on to a surface covered with cotton in order to reduce stress. Four mice were tested simultaneously, separated by black plastic walls. Mice were taken out of the apparatus when the last mouse fell. The latency to fall served as an indicator of motor performance.

#### 4.4.3. Thermal Pain Sensation Measurements

Pain sensitivity (nociception) was assessed using the hot plate test [74]. In brief, mice were placed in a Perspex cylinder on a heated stage maintained at 52 °C (NG 35150 Hot Plate; Ugo Basile, Gemonio VA, Italy). Response time was observed by heat sensitivity behavioral changes like hind paw licking, shaking, or jumping. The maximum time allowed on the plate was 30 s in order to prevent skin injury.

### 4.5. Electrophysiological Tests

Electrophysiological tests were performed on control and HFD-naive mice when mice reached 6 months of age following 18 months on either a control or HFD immediately prior to being sacrificed, as previously described [73]. Accordingly, mice were anesthetized with ketamine (100 mg/kg) and xylazine (10 mg/kg). Body temperature was maintained as warm by placing the mice on a heating mat. Temperature differences were minimized by conducting the study as soon as the anesthesia had taken effect. Electrophysiological studies were conducted on the sciatic nerve in the prone position. CMAP responses were recorded from the gastrocnemius muscle with an active needle electrode and a reference electrode placed at the center of the foot. Stimulation of the nerve was performed at the ischial notch and knee with a pair of blunt needle electrodes, with the distal cathode 10 mm proximal to the recording electrode. The ground electrode was placed between the stimulating and recording electrodes. Supra-maximal stimulation, at a range of 3–5 mA was employed, and the low and high frequency filters were set at 10 Hz and 10 kHz, respectively. To calculate the motor nerve conduction velocity (MNCV), the distance between the stimulation sites was divided by the latency difference. CMAP amplitudes were measured from the baseline to the negative peak.

### 4.6. Skin Biopsies for IENFD Measures

Hind footpa skin biopsies were collected, immersed for 6–8 h at 4 °C in Zamboni’s fixative (2% paraformaldehyde, 0.2% picric acid in 0.1 M phosphate buffer), rinsed in 30% sucrose in PBS solution overnight, cryoembedded in mounting media (OCT), and sectioned at 14 μm thick before being processed for immunohistochemistry. Sections were incubated at 4 °C for 16–24 h with rabbit anti-primary PGP9.5 antibody (1:200; Sigma-Aldrich, Singapore). Sections were then rinsed 3 times in PBS and incubated with secondary anti-rabbit AlexaFluor 488 antibody, before being rinsed and mounted. To confirm that there was no nonspecific immunoreaction, additional sections were incubated with primary or secondary antibodies alone. Fluorescent images were collected on an Olympus microscope with an ObserverZ1 imaging system (Zeiss, Oberkochen, Germany). Six sections were measured for each footpad, and the average linear density of IENF was calculated according to current guidelines [107].

### 4.7. Immunoblots

#### 4.7.1. Preparation of Plasma Protein Extracts

Plasma samples were prepared as previously described [71]. In brief, plasma samples were prepared utilizing freshly excised blood drawn from the posterior vena cava of anesthetized mice and collected into tubes containing 20 μL of 10% EDTA to prevent blood clotting. The blood was centrifuged for 10 min at 3000 rpm at 4 °C, after which the supernatant containing the plasma lipoproteins was collected and frozen at −70 °C until use.

#### 4.7.2. Preparation of Hippocampi Protein Extracts

Hippocampi samples were prepared as previously described [108]. In brief, following excision, the hippocampus of one freshly excised hemisphere was stored frozen at –80 °C. This hippocampus was then homogenized in 200 µL of the following buffer [10 mM HEPES, pH 7, which contained 2 mM EDTA, 2 mM EGTA, 0.5 mM DTT, protease inhibitor cocktail (Sigma-Aldrich P8340, Singapore), and phosphatase inhibitor cocktail (Sigma-Aldrich P5726, Singapore)]. The protein concentration was determined utilizing the BCA protein assay kit (Pierce 23225, Waltham, MA, USA). The homogenates were then aliquoted in similar protein concentrations and stored at –80 °C.

#### 4.7.3. SDS-Electrophoresis

Plasma and hippocampus samples were boiled for 10 min with 0.5% SDS and immunoblotted as previously described [71,109]. The following Abs were used: Mouse anti-IRβ (1:1000; Cell Signaling, Danvers, MA, USA), rabbit anti-IRβ (1:500; Cell Signaling, Danvers, MA, USA), rabbit anti-Akt (1:000; Cell Signaling, Danvers, MA, USA), rabbit anti-p Ser473 Akt (1:1000; Cell Signaling, Danvers, MA, USA), rabbit anti-p Thr308 Akt (1:1000; Cell Signaling, Danvers, MA, USA), mouse anti-GSK-3α/β (1:1000; Santa Cruz, Santa Cruz, CA, USA), mouse anti-p tyr216 GSK-3α/β (1:500; Santa Cruz, Santa Cruz, CA, USA), mouse anti-p Ser9 GSK-3β (1:1000; Santa Cruz, Santa Cruz, CA, USA), and goat anti-apoE (1:10,000; Millipore, Burlington, MA, USA). It is important to note that IR, Akt, and GSK-3β were assessed utilizing an antibody whose immunoreactivity corresponded to the total level of the molecule as well as the indicated phosphorylation-dependent molecule antibodies whose immunoreactivity corresponded to the indicated phosphorylated site. Membranes were scanned utilizing the ChemiDoc Touch imaging system (Bio-Rad, Hercules, CA, USA), following which blot intensity was quantified using Image Lab Software (Bio-Rad, Hercules, CA, USA). β-tubulin levels (mouse anti-β-tubulin, 1:1000; Sigma-Aldrich, Singapore) were used as gel-loading controls for hippocampi homogenates, seeing that their intensities showed similar levels in the different groups. Plasma blots were stained with 0.2% Ponceau S for the loading control. All results were normalized and presented relative to the control diet apoE3 mice.

#### 4.7.4. Blue Native Gels

For nondenaturing blue native gels, the hippocampi homogenates were run on 4%–16% gels purchased from Novex in the NativePAGE Novex Bis-Tris Gel System according to the manufacturer’s instructions, and as previously described [70]. Gels were next transferred to PVDF membranes and stained with goat anti-apoE Ab (1:10,000; Millipore, Burlington, MA, USA). The immunoblot bands were all visualized using the ECL chemiluminescent substrate (Pierce, Waltham, MA, USA), after which their intensity was visualized using the ChemiDoc Touch imaging system (Bio-Rad, Hercules, CA, USA).

### 4.8. Statistical Analysis

The experimental design consisted of 2 genotypes (apoE3 and apoE4) and 2 diets (control and HFD). All data are presented as mean ± standard error of measurement. The results of each parameter were normalized relative to the apoE3 control diet mice and analyzed via GraphPad Prism 5.3 software. Multiple groups and/or time points were analyzed utilizing two-way ANOVA, and when appropriate two-way ANOVA repeated measurements (time x groups). This was followed by a planned Bonferroni post-hoc test to determine the differential effects of diets in apoE3 and apoE4 mice. ITT measurements were analyzed by Student’s *t-*test in order to determine differences between *APOE* genotypes. Values of *p* < 0.05 were considered statistically significant. All graphs were prepared by GraphPad prism 5.3 software.

## Supplementary Materials

The following are available online at https://www.mdpi.com/1422-0067/21/4/1289/s1.

## Abbreviations

AD	Alzheimer’s disease
APP	Amyloid precursor protein
APOE	Apolipoprotein E gene
apoE4	Apolipoprotein E4 isoform
PNS	Peripheral nervous system
NMJ	Neuromuscular Junction
T2DM	Type 2 Diabetes Miletus
DPN	Diabetic peripheral neuropathy
CNS	Central nervous system
IR	Insulin Receptor
GSK-3β	Glycogen synthase kinase-3β
GS	Glycogen Synthase
HFD	High Fat Diet
TR	Targeted replacement
PBS	Phosphate buffered saline
IP	Intraperitoneally
GTT	Glucose tolerance test
AUC	Area under the curve
ITT	Insulin tolerance test
NOR	Novel Object Recognition
MNCV	Motor nerve conduction velocity
IENFD	Intra-epidermal nerve fiber density
CMAP	Compound muscle action potential
WT	Wild Type
mTORC2	Mechanistic target of rapamycin complex 2
