# Supplementary material for: Central and Peripheral Mechanisms in ApoE4-Driven Diabetic Pathology
Source: Int J Mol Sci. 2020 Feb 14;21(4):1289. doi: 10.3390/ijms21041289 (PMC7072920; doi:10.3390/ijms21041289)
Supplement: Supplementary file 1 [file ijms-21-01289-s001.pdf]

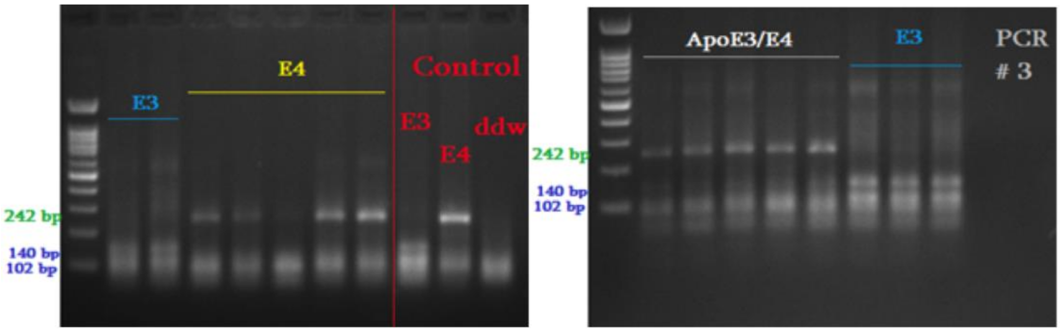

Supplementary Figure 1: Representative PCR results of apoE3/3, apoE4/4 and apoE3/4 mice. As can be seen on the left panel, the apoE3 yields a band at 140 bp and apoE4 yields a band at 242 bp. Consistent with these controls, apoE3 and apoE4 samples contain the relevant bands. As can be seen on the right panel, the apoE3/4 mice contain both the apoE3 band at 140 bp and the apoE4 band at 242 bp.
